# The Effect of *Orobanche crenata* Infection Severity in Faba Bean, Field Pea, and Grass Pea Productivity

**DOI:** 10.3389/fpls.2016.01409

**Published:** 2016-09-21

**Authors:** Mónica Fernández-Aparicio, Fernando Flores, Diego Rubiales

**Affiliations:** ^1^Institute for Sustainable Agriculture, Consejo Superior de Investigaciones CientíficasCórdoba, Spain; ^2^INRA, UMR1347 AgroécologieDijon, France; ^3^Escuela Técnica Superior de Ingeniería – Universidad de HuelvaPalos de la Frontera, Spain

**Keywords:** parasitic weed damage, legume, broomrape, resource allocation, weed threshold density

## Abstract

Broomrape weeds (*Orobanche* and *Phelipanche* spp.) are root holoparasites that feed off a wide range of important crops. Among them, *Orobanche crenata* attacks legumes complicating their inclusion in cropping systems along the Mediterranean area and West Asia. The detrimental effect of broomrape parasitism in crop yield can reach up to 100% depending on infection severity and the broomrape-crop association. This work provides field data of the consequences of *O. crenata* infection severity in three legume crops, i.e., faba bean, field pea, and grass pea. Regression functions modeled productivity losses and revealed trends in dry matter allocation in relation to infection severity. The host species differentially limits parasitic sink strength indicating different levels of broomrape tolerance at equivalent infection severities. Reductions in host aboveground biomass were observed starting at low infection severity and half maximal inhibitory performance was predicted as 4.5, 8.2, and 1.5 parasites per faba bean, field pea, and grass pea plant, respectively. Reductions in host biomass occurred in both vegetative and reproductive organs, the latter resulting more affected. The increase of resources allocated within the parasite was concomitant to reduction of host seed yield indicating that parasite growth and host reproduction compete directly for resources within a host plant. However, the parasitic sink activity does not fully explain the total host biomass reduction because combined biomass of host–parasite complex was lower than the biomass of uninfected plants. In grass pea, the seed yield was negligible at severities higher than four parasites per plant. In contrast, faba bean and field pea sustained low but significant seed production at the highest infection severity. Data on seed yield and seed number indicated that the sensitivity of field pea to *O. crenata* limited the production of grain yield by reducing seed number but maintaining seed size. In contrast, the size of individual parasites was not genetically determined but dependent on the host species and resource availability as a consequence of competition between parasites at increasing infection severities.

## Introduction

Broomrape weeds (*Orobanche* and *Phelipanche* species) are root-holoparasitic plants that possess extreme competitive ability against the crop. Rather than to compete with crops for field resources, their haustorial cells penetrate crop roots to directly divert water and nutritive resources (Parker and Riches, 1993). Broomrape weeds attack dicotyledonous crops along Mediterranean, central and Eastern Europe, and Asia (Parker, 2009). *Orobanche crenata* Forsk is a major constraint for grain and forage legume on over 4 Mha of the Mediterranean area (Parker, 2009). The increasing interest on sustainable agriculture promotes the cultivation of legumes as a tool of ecological optimization of resource use and promotion of pest resilience in cropping systems. Due to the severe effects of *O. crenata* parasitism in the host crop, and the high persistence of parasitic seedbank in agricultural soils it has been the cause of abandonment of legume cultivation in important cropping areas (Mesa-Garcia and Garcia-Torres, 1984; Sauerborn, 1991; Parker and Riches, 1993; Rubiales et al., 2009).

Many of the broomrape traits such as achlorophyllous nature, underground parasitism, the physical and metabolic overlap with the crop, or lack of functional roots, reduce the efficiency of conventional programs in weed management aimed to their control (Fernández-Aparicio et al., 2016). In order to achieve efficient control, broomrape parasitism should be targeted at different fronts by an integrated management strategy (Kebreab and Murdoch, 2001). Infection severity in broomrape strongly depends on environmental factors such as temperature and parasitic seedbank density (Linke et al., 1991; Eizenberg et al., 2005). For the determination of thresholds of weed density above which it is profitable to apply control measurements, equations correlating yield losses to broomrape infection severity are needed (Garcia-Torres et al., 1996) but their determination is spare in the majority of crops affected. No previous work has estimated the effect of *O. crenata* infection in field pea and grass pea. The *O. crenata* effect on faba bean productivity was estimated in pots by Manschadi et al. (1996) at three successively increased seedbank densities, in which even at the lowest seed density, a high number of broomrapes was attached per host plant, while at the other two increased densities the parasite number was observed so high that lead to faba bean death before maturity. In contrast, a field study by Mesa-Garcia and Garcia-Torres (1984) provided an accurate prediction system for expected damage by studying the range of infections normally found in the field. Caution should be exercised in the extrapolation of host and parasite growth in pots to real farming conditions, as the rapid saturation in parasite-carrying capacity occurring in pots complicates the determination of the host response curves related to infection severity.

Our study was conducted to determine the consequences in crop productivity of *O. crenata* parasitism at successively increasing infection severities in three highly susceptible legume crops: faba bean (*Vicia faba* L.), field pea (*Pisum sativum* L.), and grass pea (*Lathyrus sativus* L.). The experimentation was carried out in field conditions with a natural parasitic seed bank in south of Spain, an area where *O. crenata* is endemic attacking legume crops.

## Materials and Methods

### Plant Material

One susceptible cultivar of each of the three species faba bean (cv. Prothabon), field pea (cv. Messire), and grass pea (cv. Lisa) were chosen to evaluate their response to increasing levels of *O. crenata* parasitism. A natural occurring parasitic seedbank of *O. crenata* was infesting the field used in the experiments.

### Site and Experimental Design

The experimental work was carried out in Córdoba, (Alameda del Obispo Farm), southern Spain, on a deep loam soil (typic xerofluvent). The precrop on the experimental site was faba bean- wheat- field pea rotation. Average (30 years) annual precipitation and air temperature in the area were 536.0 mm and 17.6°C, respectively, with maximum and minimum daily air temperature of 46.6°C and -7.8°C. Each host species was laid out in 15 × 4 m^2^ experimental plots, in a complete randomized block design with three replicates. Each crop was hand sown in November, each plant separated 50 cm apart inside rows with 50 cm distance between rows and 5 cm sowing depth. Legumes were rain feed, and hand weeding of weeds other than broomrape was carried out when required.

The field was deliberately chosen by its heterogeneous distribution of *O. crenata* seed bank indirectly observed in previous seasons by a patchy distribution of the parasite in susceptible legume plants. Consequently, host plants inside each plot suffered variation of infection severity measured as number of emerged broomrapes per host plant. At the end of cultivation cycle, data on emerged broomrapes ranging from 0 to 11 parasites per host plant was taken in 750 host plants. All broomrapes emerged per host plant were extracted from the host root by a gentle pull and counted. Host and parasitic tissue was collected per each host plant and carried to the laboratory. Samples were dried at 80°C during 48 h and each biomass compartment weighed independently per host plant.

### Calculation and Statistics

*O. crenata* infection severity was estimated per individual host plant in each crop species as **number of emerged broomrapes per plant**. The distribution of dry matter within the host–parasite complex was determined by recording five parameters per host plant: **combined biomass** (host and broomrape biomass), **aboveground host dry matter,** **host** **reproductive dry matter** (host seeds), **host** **vegetative dry matter** (aboveground minus reproductive dry matter), and **cumulative broomrape dry matter** (total broomrape dry biomass per host plant). In addition, for field pea plants, **number of seeds** per plant was measured in each sampled plant.

Several parameters were calculated to characterize the effect of *O. crenata* parasitism in each crop. First, host **reproductive index** was determined taking into account only host tissue and it was calculated for each plant as the ratio between host reproductive dry matter and total aboveground host dry matter. Additional parameters were calculated to determine the biomass partitioning within the host–parasite complex: **relative host reproductive weight** (percentage of the combined biomass allocated into the host seeds); **relative parasitic weight** (percentage of the combined biomass allocated into the parasite); **relative weight of total sinks** (percentage of the combined biomass allocated into host seeds and in the parasite). In addition the **individual *O. crenata*** **weight** was estimated as parasitic biomass sustained by each host plant averaged by the number of parasites per plant. For field pea, the **individual** **seed weight** was calculated as host reproductive biomass averaged by the number of seeds.

Statistical analysis was performed using SAS (R) 9.3 (SAS Institute Inc.). Arcsine square root transformations of the data which did not meet the conditions of normality and homogeneous variance were performed to conform to the assumptions of analysis of variance (ANOVA). ANOVA was conducted on biomass data using a randomized design, to test for the significance of the infection severity.

In each host species, infection severity was regressed against all components in the system response to parasitism: total aboveground host biomass, host vegetative biomass, host reproductive biomass, number of seeds, average seed weight, combined biomass, relative parasitic weight, relative host reproductive sink, and relative weight of total sinks. In addition, number of parasites was regressed against individual parasitic weight average.

## Results And Discussion

**FIGURE 1:**
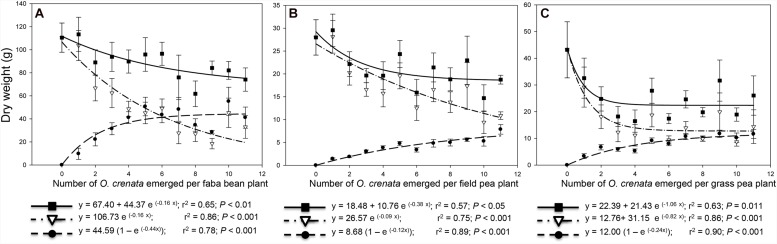
**Relationship between total dry weight and infection severity measured as *O. crenata* emergence per host plant.** Dry weight of infected system (combined host+ *O. crenata*; ■), host dry weight (▽), cumulative *O. crenata* dry weight (●). **(A)** faba bean, **(B)** field pea; **(C)** grass pea.

**FIGURE 2:**
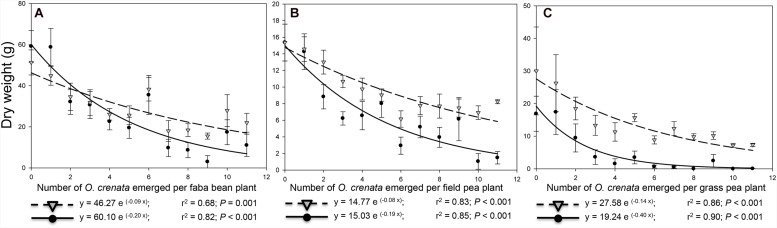
**Relationship between total dry weight and infection severity measured as *O. crenata* emergence per host plant.** Host vegetative dry matter (leaves and stems; ▽), Host reproductive dry matter (host seeds; ●). **(A)** faba bean, **(B)** field pea; **(C)** grass pea.

In parasite-free plants, the average **aboveground dry matter** and **reproductive index** (ratio host seeds to aboveground host dry matter) were, respectively, 110.5 g and 53.6% for faba bean, 28.0 g and 53.5% for field pea and 43.2 g and 35.0% for grass pea plant (**Figures 1** and **Figures 2**). Our results are in agreement with previous studies showing that low reproductive index is characteristic of grass pea species (Siddique et al., 1996). During parasitism, broomrape plant acts as additional sink withdrawing water and assimilates from host vascular system. In the three legume species studied, aboveground host dry matter was consistently reduced by *O. crenata* parasitism in an infection severity-dependent fashion. Correlations between aboveground dry matter in the host and infection severity have been previously observed high in faba bean-*O. crenata* (Mesa-Garcia and Garcia-Torres, 1984), while was low or not significantly correlated in other crop-broomrape associations such as sunflower-*O. cernua* (Garcia-Torres et al., 1996), tobacco-*O. cernua* (Hibberd et al., 1998), and tomato-*P. ramosa* (Mauromicale et al., 2008). In our field data, the relation between aboveground host dry matter reduction and infection severity was best fitted to exponential functions where the downward slide began at low parasite densities and intensified at a biomass inhibition rate of 0.16, 0.09, and 0.82 g/g indicating that parasitism reduced aboveground host dry weight in faba bean, field pea, and grass pea by 16, 9, and 82% for weight unit, respectively. The severe reduction in grass pea biomass was linear up to three parasites per plant, after which there was not further decrease in host dry matter with increased parasite densities. Similar pattern was previously described in tomato infected with increased densities of the broomrape species *P. aegyptiaca* (Barker et al., 1996). In our field study, the infection severity with half maximal inhibitory performance was predicted as 4.5, 8.2, and 1.5 parasites per faba bean, field pea, and grass pea plant, respectively. Tolerance to broomrape weeds is defined by the capacity of a given crop genotype to endure broomrape infection with low productivity losses (Fernández-Aparicio et al., 2016). Our field data show that at any given level of infection severity, grass pea is the species less tolerant to *O. crenata* parasitism except at the highest parasite density, in which each legume species only achieved 30.0, 38.0, and 33.0% of the total aboveground dry matter of their respective faba bean, field pea, and grass pea uninfected counterparts (**Figures 1A–C**).

**Figure 2** shows that the *O. crenata*-induced inhibition rate was more intense in reproductive than in vegetative dry matter measured at final harvest. Reductions in the reproductive index were previously observed in *O. minor*-red clover and *O. crenata*-faba bean by Mesa-Garcia and Garcia-Torres (1984), Manschadi et al. (1996), and Lins et al. (2007). The parasite density with half maximal inhibitory performance in faba bean, field pea, and grass pea was, respectively, predicted as 7.6, 8.2, and 4.8 parasites per plant for host vegetative dry matter and predicted as 3.5, 3.7, and 1.7 parasites per plant for host reproductive dry matter. Mesa-Garcia and Garcia-Torres (1984) predicted that four *O. crenata* per plant was the average infection severity responsible for the reduction of faba bean seed yield by half, being similar to our predictive system for faba bean seed reduction. It has been previously suggested that seed yield losses induced by *O. crenata* can reach up to 100% (Sauerborn, 1991). In grass pea, the seed yield was negligible at severities higher than four parasites per plant. In contrast, at the highest infection severity (11 broomrapes per plant) faba bean and field pea sustained a low but significant seed production.

The **cumulative dry biomass** of all broomrapes infecting one single plant was highly related with infection severity (**Figure 1**). Exponential rise functions were fitted to these data suggesting that biomass accumulation in parasites rise quickly at low parasite densities and the rate of increase starts to slow at densities higher than five parasites per plant regardless host species. High correlation between broomrape number and broomrape dry weight accumulated per host plant was previously observed by Mesa-Garcia and Garcia-Torres (1984), Barker et al. (1996), Garcia-Torres et al. (1996) but differed from that reported by Manschadi et al. (1996) and Hibberd et al. (1998). In faba bean, the maximum accumulation of parasitic biomass (55.18 ± 12.27) was higher than that observed in field pea (7.86 ± 1.04) and in grass pea (11.6 ± 1.67). For faba bean and grass pea the maximum accumulated parasitic dry weight was not significantly different from the seed yield produced by uninfected plants. On the contrary, for field pea the maximum parasitic dry matter was significantly lower than the dry matter of seeds in uninfected field pea plants.

Living as obligated holoparasites, broomrapes are deprived of autotrophy and in consequence infected host plants are responsible for capture and synthesis of resources used for the biomass construction of the overall parasitic plant-host plant complex. For some crop-broomrape associations, combined biomass is equivalent to that developed by uninfected crops being the biomass loss in the host equivalent to that accumulated by the parasite. This performance suggests that damage in the crop is directly attributed to the parasitic sink activity for nutrient withdrawal. However, the damage induced by parasitic weeds in other crops extends beyond assimilate diversion. In those cases, the parasitic weed displays a pathogenic-like nature promoting negative effects on the crop photosynthetic machinery and hormonal balance (Stewart and Press, 1990; Barker et al., 1996; Manschadi et al., 1996; Hibberd et al., 1998; Mauromicale et al., 2008). Therefore, we studied whether in our field conditions, *O. crenata* parasitism impairs the capacity of faba bean, field pea, and grass pea as autotrophic sources of energy and nutrients regardless the final sink allocation within the plant-parasitic plant complex. The productivity of the infected system (**total** **host**–**parasite combined dry weight)** was slightly lower than the total biomass developed by uninfected plants (**Figure 1**). Similar results have been reported for *O. cernua*-tobacco association (Hibberd et al., 1998). The inhibition rate of combined biomass was more intense in grass pea (1.06 g/g) than in field pea (0.38 g/g) and faba bean (0.16 g/g), reaching asymptotic values in which each legume species achieved 60, 67, and 67% of the total biomass of their respective uninfected counterparts. These results suggest that the reduction of biomass allocated into the host was not fully explained by the *O. crenata* sink activity.

**FIGURE 3:**
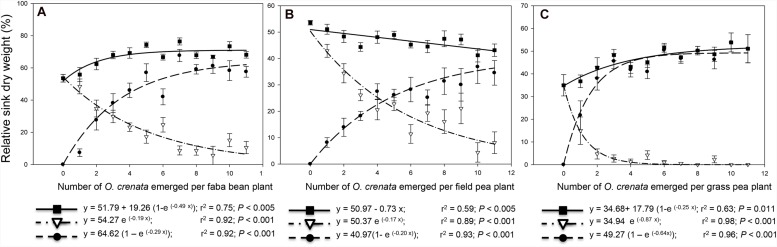
**Relationship between relative sink weight (ratio of sink weight to total complex dry weight) and infection severity measured as *O. crenata* emergence per host plant.** Combined sink (host seed + *O. crenata*) relative weight (■), host seed relative weight (▽), *O. crenata* relative weight (●). **(A)** faba bean, **(B)** field pea; **(C)** grass pea.

In order to determine whether there is a trade-off between the resources allocated into reproductive versus parasite sink through the range of infection severity observed, we studied the **combined weight** **of sinks** (host seed plus parasite dry weights) and the **relative weight** **of** **combined sink** (ratio between combined sink weight to total aboveground biomass) at increased infection severities. In faba bean and grass pea, the combined weight of sinks equals the reproductive sink (seed weight) in uninfected plants regardless the level of infection (data not shown). Similar results were reported by Manschadi et al. (1996). By contrast, in field pea the combined sink weight was reduced up to 44.9% with respect to reproductive weight in uninfected plants. This reduction was a consequence of relatively larger host seed inhibition than the concomitant gain in parasitic dry weight as described above. This reduction occurred at a rate of 0.64 g/g up to infection severity of two parasites per plant but remained constant thereafter. **Figure 3** shows the relative distribution of dry matter into sinks within the host-*O. crenata* complex. In faba bean and grass pea but not in field pea, the relative weight of combined sinks was significantly higher than the reproductive index and in the uninfected system and this difference was related with infection severity (ANOVA, *p* < 0.001; **Figure 2**). This performance was due to a larger decrease in dry weight of vegetative compartment at final harvest in relation to a constant combined sink weight suggesting that as the infection severity increases, higher levels of leaf reserves needs to be remobilized to attend the cost of combined sink biomass construction versus that occurred to build the equivalent seed biomass in uninfected plants. It was previously described that in the system *O. cernua*-tobacco, the host leaf area is not altered during *Orobanche* infection but the specific leaf area increases at late stages in the infection process indicating remobilization of leaf reserves (Hibberd et al., 1998).

In order to compare the strength of the parasitic sink for assimilate allocation in each host species, we studied the **relative weight of parasites** (ratio of accumulation of parasitic dry weight relative to combined biomass). For faba bean and grass pea the relative parasitic weight increased rapidly at low infection severity but it found an upper limit established around five parasites per plant beyond which it did not change significantly suggesting that a maximum percentage of dry matter within the host–parasite complex can be derived to the parasite regardless the infection severity (**Figures 3A,C**). However, in field pea, the increase rate in relative parasitic weight was slightly lower but more sustained through a broader range of parasite severity, reaching the upper limit at higher levels of infection (**Figure 3B**). The parasite relative weight ranged from 7.4 (1 parasite per plant) to 57.6% (11 parasites per plant) in faba bean; from 8.2 (1 parasite per plant) to 41.6% (11 parasites per plant) in field pea; and from 21.8 (1 parasite per plant) to 51.0% (11 parasites per plant) in grass pea. These data seems to indicate that less assimilates is available to *O. crenata* plants when they are attached to field pea roots. In the case of grass pea, parasitic relative weight equals combined sink relative weight at parasites densities higher than 4, due to the total substitution of seed yield by parasite dry matter. This effect was not observed in faba bean and field pea due to low but sustained seed production in the host. The proportion of resources allocated within the parasite was concomitant to reduction of host seed yield indicating that parasite growth and host reproduction compete directly for resources within a host plant. Host seed relative weight decreased at greater parasite densities according to exponential decay functions from 48.5 (1 parasite per plant) to 10.6% (11 parasites per plant) in faba bean, from 42.9 (1 parasite per plant) to 8.1% (11 parasites per plant) in field pea; and from 15.0 (1 parasite per plant) to 0.1% (11 parasites per plant) in grass pea (**Figure 3**).

**FIGURE 4:**
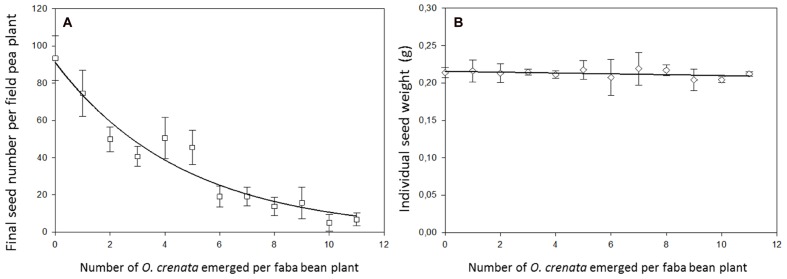
**Relationship between seed yield components in faba bean and infection severity. (A)** Number of harvested seeds per host plant (*y* = 9107 e^(-0.21^*^x^*^)^, *r*^2^ = 0.93, *P* < 0.0001), **(B)** Individual seed weight (*y* = 0.21-0.0006*x*), *r*^2^ = 0.16, *P* = 0.19).

Data recorded in field pea on seed yield and number of seeds per plant appears to suggest that field pea adjusts grain yield components in response to *O. crenata* parasitism by reducing number of seeds per host plant but maintaining constant the individual seed weight (**Figure 4**). Similarly *O. crenata* infection reduced the seed number but not the average seed unit weight in faba bean (Mesa-Garcia and Garcia-Torres, 1984). In our work, uninfected field pea plants produced an average of 93.4 ± 11.9 seeds per plant which was inhibited at a rate of 21% by increasing levels of *O. crenata* infection. The parasite density with half maximal inhibitory performance for seed number was predicted as 3.3 parasites per plant and at the highest parasite density, pea plants only produced 7.3% seeds of their uninfected counterparts (**Figure 4A**). The average weight of individual field pea seeds was 0.22 g per seed and was independent on the level of infection indicating that it was genetically controlled regardless the availability of resources (**Figure 4B**). This may be part of a host survival strategy to parasitic plant infection, where field pea concentrates its resources to a smaller number of viable seeds being this strategy also observed in legumes to survive drought (Gusmao et al., 2012). Those results are in agreement with the Smith and Fretwell (1974) model which establishes that seed individual weight is genetically determined and less variable in response to changes in resource availability than seed number which is directly regulated by the resource availability averaged by the genetically determined seed individual weight. Competition for pollinators between host and *O. crenata* inflorescences could not have a role in the reduction of number of seeds in field pea infected plants, because field pea is an autogamous species, however, it should be considered in other parasitic plant-host plant interactions when both partners in the host–parasite complex are allogamous and their flowering stage is synchronized.

**FIGURE 5:**
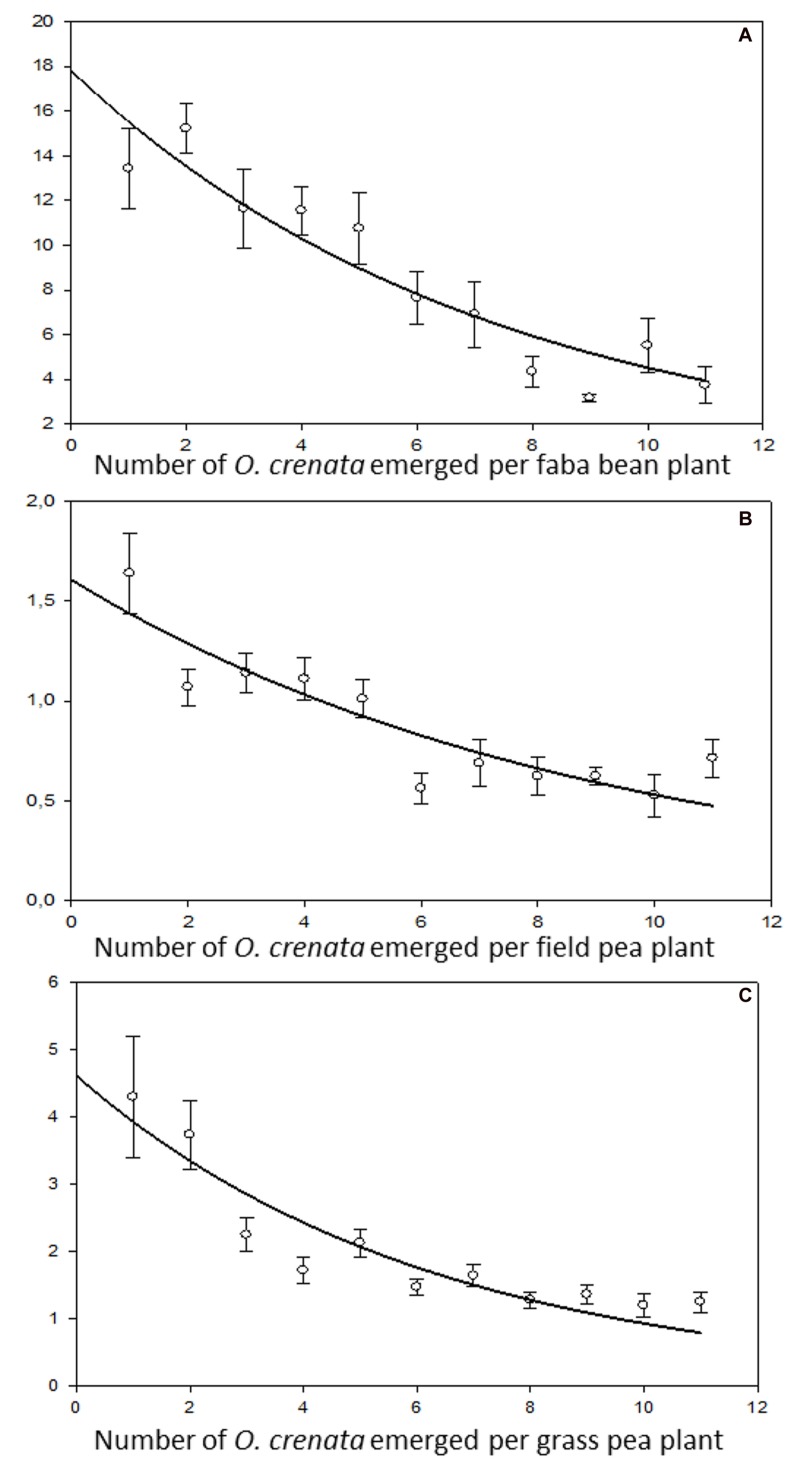
**Relationship between average dry weight of individual *O. crenata* plant and infection severity measured as *O. crenata* emergence per host plant. (A)** Faba bean (*y* = 17.82 e^(-0.14^*^x^*^)^, *r*^2^ = 0.89, *P* < 0.0001), **(B)** field pea (*y* = 1.61e^(-0.11^*^x^*^)^, *r*^2^ = 0.80, *P* = 0.0002), **(C)** grass pea (*y* = 4.62 e^(-0.16^*^x^*^)^, *r*^2^ = 0.85, *P* < 0.0001).

The regulation model of seed production in response to differing resource availability contrasted with the regulation model of resource distribution observed in the parasites attached to the same host plant. The size of individual parasites (indirectly estimated as **average weight of individual parasite)** was not genetically controlled but dependent on the host species. Individual parasite weight was bigger in faba bean (at low parasite densities the individual weight per parasite was 10.34 ± 0.22 g) than in grass pea (2.36 ± 0.41 g) followed by field pea (1.06 ± 0.09 g). In addition, parasite individual weight was very plastic in response to resource availability. A negative relation between *O. crenata* individual weight and parasite density was observed, suggesting competition between individual parasites feeding in a single host plant at high parasite densities (**Figure 5**). Our observations agreed to those observed by Barker et al. (1996), Manschadi et al. (1996), and Hibberd et al. (1998) in various crop-broomrape associations.

## Conclusion

Studies of competitive relations between parasitic weeds and its host crops are important in order to calculate intervention thresholds of control measurements essential in integrated pest management programs. Data of how *Orobanche* affects the growth of attacked crops is not always available or applicable. Most available studies in broomrape-crop associations have been performed in pots which reflect imperfectly the consequences of broomrape parasitism in the crop. We have contributed to breach this gap of knowledge in legume crops by characterizing in field *O. crenata* growth and the consequences of its parasitism in faba bean, field pea, and grass pea, three of its most preferred host crops.

## Author Contributions

MF-A designed, implemented the study and collected the data. FF analyzed the data. MF-A and FF interpreted the data. MF-A wrote the manuscript. FF and DR revised the manuscript. DR contributed with materials and laboratory equipment.

## Conflict of Interest Statement

The authors declare that the research was conducted in the absence of any commercial or financial relationships that could be construed as a potential conflict of interest.
